# New predictions of ^137^Cs dynamics in forests after the Fukushima nuclear accident

**DOI:** 10.1038/s41598-019-56800-5

**Published:** 2020-01-08

**Authors:** Shoji Hashimoto, Naohiro Imamura, Shinji Kaneko, Masabumi Komatsu, Toshiya Matsuura, Kazuya Nishina, Shinta Ohashi

**Affiliations:** 1 Department of Forest Soils, Forestry and Forest Products Research Institute, Tsukuba, Ibaraki 305-8687 Japan; 2 Graduate School of Agricultural and Life Sciences, The University of Tokyo, Bunkyo-ku, Tokyo 113-8657 Japan; 3 Kansai Research Center, Forestry and Forest Products Research Institute, Fushimi, Kyoto 612-0855 Japan; 4 Department of Mushroom Science and Forest Microbiology, Forestry and Forest Products Research Institute, Tsukuba, Ibaraki 305-8687 Japan; 5 Department of Forest Management, Forestry and Forest Products Research Institute, Tsukuba, Ibaraki 305-8687 Japan; 6 Center for Regional Environmental Research, National Institute for Environmental Studies, Tsukuba, 305-8506 Japan; 7 Department of Wood Properties and Processing, Forestry and Forest Products Research Institute, Tsukuba, Ibaraki 305-8687 Japan

**Keywords:** Ecology, Environmental sciences

## Abstract

Most of the area contaminated by the Fukushima Daiichi Nuclear Power Plant accident is covered by forest. In this paper, we updated model predictions of temporal changes in the ^137^Cs dynamics using the latest observation data and newly provided maps of the predicted ^137^Cs activity concentration for wood, which is the most commercially important part of the tree body. Overall, the previous prediction and latest observation data were in very good agreement. However, further validation revealed that the migration from the soil surface organic layer to the mineral soil was overestimated for evergreen needleleaf forests. The new prediction of the ^137^Cs inventory showed that although the ^137^Cs distribution within forests differed among forest types in the first 5 years, the difference diminished in the later phase. Besides, the prediction of the wood ^137^Cs activity concentrations reproduced the different trends of the ^137^Cs activity concentrations for cedar, oak, and pine trees. Our simulation suggests that the changes of the wood ^137^Cs activity concentration over time will slow down after 5–10 years. Although the model uncertainty should be considered and monitoring and model updating must continue, the study provides helpful information on the ^137^Cs dynamics within forest ecosystems and the changes in wood contamination.

## Introduction

Forests represent the major land use of the area contaminated by the Fukushima Daiichi Nuclear Power Plant (FDNPP) accident^1^, covering approximately 70% of the area. Because of its long half-life, ^137^Cs contamination persists longer than that of other nuclides (e.g., ^134^Cs or ^131^I), and the dynamics of ^137^Cs in contaminated forests is of great concern.

According to the studies conducted in Europe after the Chernobyl nuclear accident and as confirmed by studies conducted in Japan after the FDNPP accident, the ^137^Cs deposited on forests is expected to circulate within the forest ecosystems. At the time of the fallout, needles/leaves, branches, and stems trapped ^137^Cs, and a part of the fallout was deposited directly on the soil surface^2,3^. However, these distributions drastically change with time, particularly within a couple of years^3^. During that early phase, the major migration flux is from the tree to the soil surface. Specifically, the trapped ^137^Cs moves from the tree to the soil surface via rainfall and by the shedding of dead needles/leaves. Eight years have passed since the FDNPP accident, and many studies have demonstrated the dynamic migration and recycling of ^137^Cs within forests in Japan^2–5^.

The dynamics of ^137^Cs within forests represent information that is important because it not only captures the situation of the forest contamination but also clarifies our understanding and evaluation of the changes in the ambient dose rate in forests. Furthermore, this critical information can be provided to local peoples and to authorities of forest management so that they can implement effective countermeasures. In addition, although the proportion of ^137^Cs in wood to the total inventory is very small (less than a few percentage points)^3,4^, wood is an important natural resource and is used for timber and as logs for mushroom cultivation. Thus, the ^137^Cs activity concentration in wood both currently and in the future are of great concern for people in terms of external and internal exposure risks and the regulation of wood use.

Modelling is a powerful tool to holistically track the dynamics of radionuclides in forest ecosystems^6^. After the Chernobyl nuclear accident, many models were developed^7,8^, and an intercomparison project was conducted to assess model performance and uncertainty^7^. Similarly, after the FDNPP accident, several modelling studies were conducted to understand the ^137^Cs dynamics and evaluate the ability and uncertainty of the obtained results^9–14^. For instance, Nishina *et al*. developed a new radionuclide model by combining the forest growth model and the soil carbon dynamics model and validated the model using 7-year monitoring data^10,11^. Furthermore, Ota *et al*. developed a model to track the detailed ^137^Cs dynamics in forest soils^13^. Calmon *et al*. also simulated the tree-to-soil surface migration in the early phase^14^, whereas Mahara *et al*. predicted the future tree uptake of soil ^137^Cs [ref. ^12^].

One of the first modelling studies that were carried out after the FDNPP accident was the spatio-temporal prediction of ^137^Cs dynamics by Hashimoto *et al*.^9^ Specifically, the authors parameterized a radionuclide model—the RIFE1 model—which was developed after the Chernobyl accident, using data observed in forests during the first 2 years following the FDNPP accident; the authors then coupled the simulated results with an air-borne survey map and predicted the spatio-temporal prediction of the ^137^Cs distributions within forests. Monitoring continued after the previous prediction, and now the latest observation data enable us to verify/validate and update the previous prediction.

The main goals of the present study are to (1) verify the previously predicted dynamics, based on the latest observation data, (2) update the prediction of the temporal changes of ^137^Cs dynamics within the contaminated forests by parameterizing the model with the latest observation data, and (3) predict the spatio-temporal changes in ^137^Cs activity concentration in wood. To that end, we modified the RIFE1 model and updated the model parameters using the latest observation data; then, we simulated the ^137^Cs dynamics within forests. We further estimated the activity concentration of ^137^Cs in wood by combining the output of the modified RIFE1 model with a wood growth model. We finally coupled the temporal simulation with the spatial data obtained by an air-borne survey.

## Results

**Figure 1:**
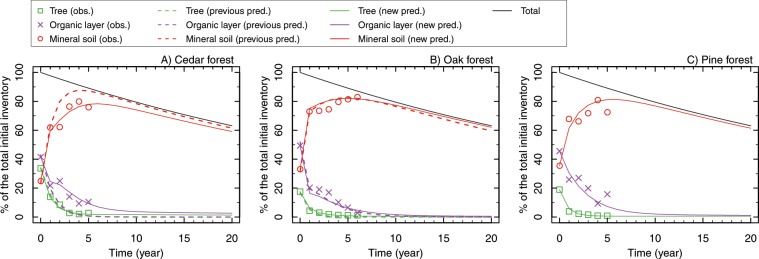
Temporal changes in the ^137^Cs distributions in cedar (**A**), oak (**B**), and pine (**C**) forests (Otama site). The solid lines are new predictions (this study), and the dashed lines are from Hashimoto *et al*.^9^, which are based on the observation data in 2011 and 2012.

A new prediction of the temporal changes in the ^137^Cs distributions within forests was compared with the latest observation data and the previous prediction study (Fig. 1, and see Supplementary Fig. S1 for only the previous prediction). The overall ^137^Cs dynamics seen in the observation data indicate that the ^137^Cs captured in the tree canopy and soil surface organic layer migrated to the mineral soil (soils with minerals, humus, and organic matter beneath the soil surface organic layer) in the first 5 years, and the mineral soil now serves as the largest ^137^Cs reservoir. Both the new and the previous predictions adequately captured the overall ^137^Cs dynamics within the forests. The previous prediction for the tree compartments (needles/leaves, branches, woods, and barks) agreed surprisingly well with the observation data for both forests (green broken lines) and adequately predicted the ^137^Cs inventory in the organic layer and in the mineral soil in the oak forest (purple and red broken lines). However, differences were found in the organic layer and the mineral soil in the cedar forest: the observed inventory in the organic layer dropped quickly from 2011 and 2012. However, the decrease in the ^137^Cs in the organic layer subsequently slowed, and the previous prediction overestimated the decrease in the organic layer fraction. As a result, the previous prediction overestimated the migration from the soil surface organic layer to the mineral soil. The new prediction adequately reproduced the retention of ^137^Cs in the organic layer and indicated that the amount of ^137^Cs in the organic layer will become very small and comparable to the inventory in the tree compartments in all forests after 10 years.

**Figure 2:**
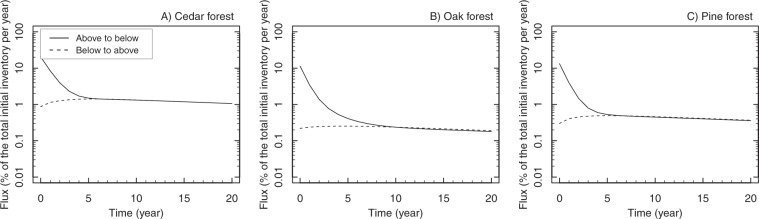
Temporal changes in the ^137^Cs flux from tree to soil (above to below: solid lines) and tree uptake (below to above: dashed lines) in cedar (**A**), oak (**B**), and pine (**C**) forests (Otama site).

To quantify the ^137^Cs dynamics within the forests, the simulated ^137^Cs fluxes from the aboveground tree compartments to the forest floor and from the soil compartments to the tree compartments (e.g., root uptake) are shown in Fig. 2. It is important to note that the initial observation data used for the simulation were first taken in August and September 2011, and hence, the migration of ^137^Cs in the first 5 months (e.g. initial capture by tree canopy in March 2011 and its release in that period) was not captured in this simulation. In 2011, approximately 10–20% of the total initial inventory moved from the tree to the forest floor in all forests, and the flux rapidly decreased in the next 5–10 years. On the other hand, the simulated root uptake slowly increased in the first couple of years, and the upward flux and downward flux were almost balanced after 5–10 years. The two fluxes slowly decreased with the radioactive decay of ^137^Cs in the later phase of the simulation, and the flux was approximately 0.2–1% of the total initial inventory in the later phase. Because the oak and pine forests were mixed forests, the fluxes were divided for each tree species (Supplementary Fig. S2). The simulated flux converged more quickly for pine trees (3 years) than for oak trees (5–7 years), and, as seen by the continuously rising ^137^Cs activity concentration in oak wood^3,15^, the uptake flux was greater than the downward flux in oak trees.

**Figure 3:**
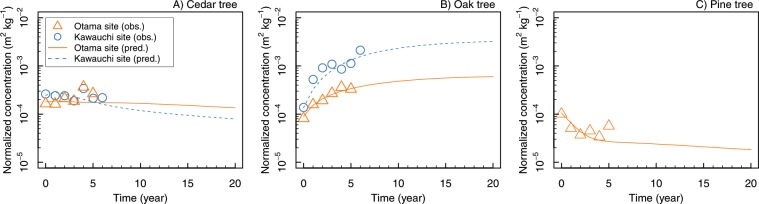
Estimated temporal changes in the normalized ^137^Cs activity concentration in wood for cedar (**A**), oak (**B**), and pine (**C**) trees. The lines are predictions. Please note that the model for cedar and oak was parameterized with data obtained from both Otama and Kawauchi sites (see the Methods section), respectively. There was no observation data for pine from the Kawauchi site.

The observed ^137^Cs activity concentration in cedar and oak wood showed different trends, and the modelling well reproduced the overall trends (Fig. 3): the concentration fluctuated for both forests; however, the concentration for cedar was stable or slightly increasing in Otama, while that for oak rapidly increased in the first 3 years and then the increasing trend weakened. The concentration of pine decreased in the first 3 years and then steadied. Although the magnitude differed for oak trees, there were similar trends between the Otama and the Kawauchi sites.

**Figure 4:**
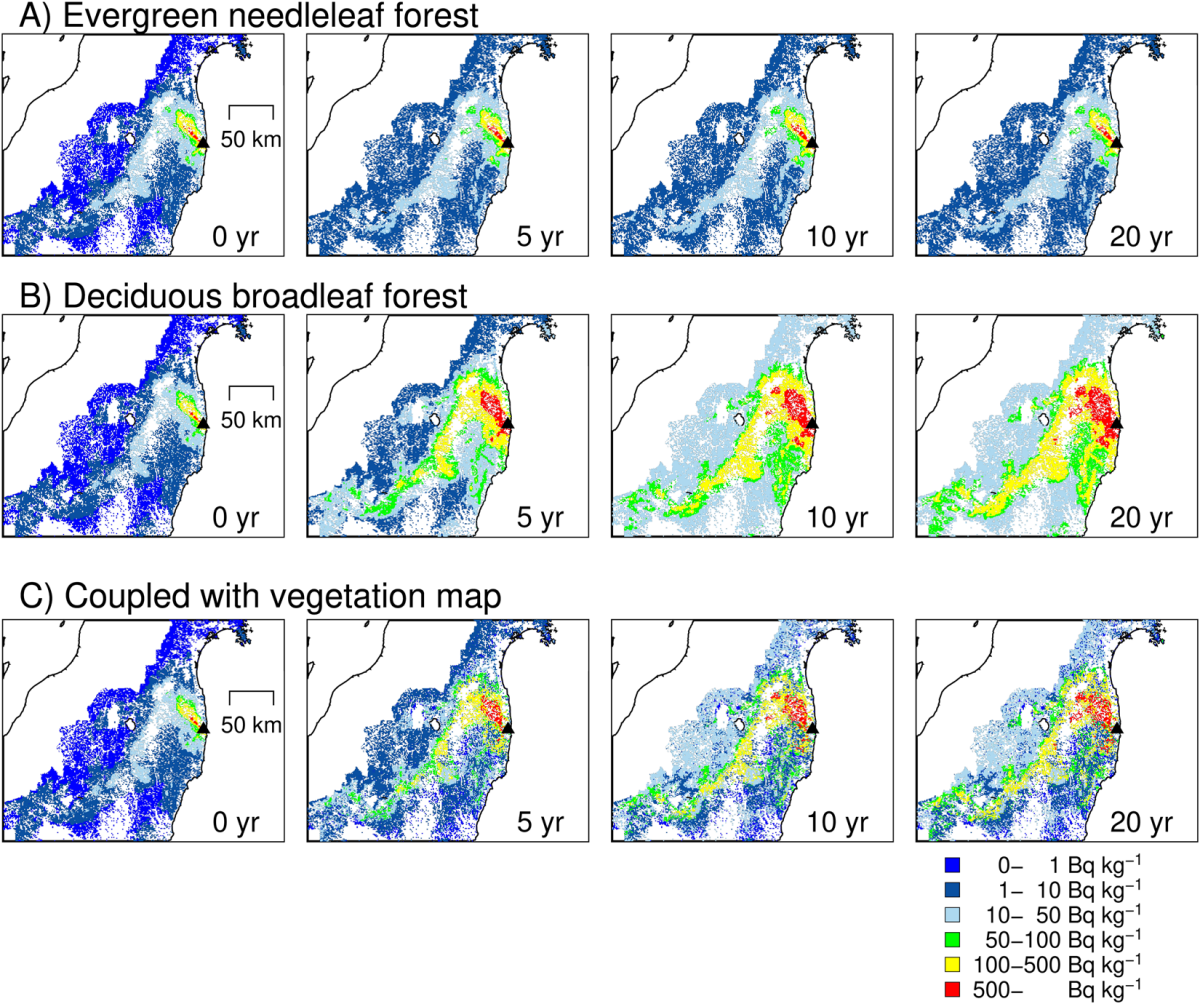
Predicted ^137^Cs activity concentration in wood following the FDNPP accident. Top (**A**) evergreen needleleaf forest (trajectory based on Otama cedar trees), middle (**B**) deciduous broadleaf forest (trajectory based on Kawauchi oak trees), and bottom (**C**) coupled with the vegetation map. The above two maps assume that all forests are exclusively evergreen needleleaf forests (top) or deciduous broadleaf (middle) forests, respectively. The bottom maps were generated by combining the above two maps using a vegetation map. The maps were created using the Generic Mapping Tools version 5 (http://gmt.soest.hawaii.edu/).

The temporal simulation of the ^137^Cs activity concentration was coupled with an air-borne-based ^137^Cs map and a vegetation map (Supplementary Fig. S3), and the prediction maps of the ^137^Cs activity concentration in wood were generated (Fig. 4). To provide a conservative assessment, we adopted the temporal trends with higher normalized concentrations: the temporal trend of cedar in the Otama site for the evergreen needleleaf forests and oak in the Kawauchi site for the deciduous broadleaf forests. The map (Fig. 4) shows the spatio-temporal changes of the ^137^Cs activity concentration in wood, ranging from 0–1 Bq kg^−1^ to more than 500 Bq kg^−1^: the upper two rows illustrate the ^137^Cs activity concentration for the evergreen needleleaf forests and deciduous broadleaf forests, and the bottom row presents the results after coupling with the vegetation map (e.g., the grid with the needleleaf forest is coloured with the result from cedar). The ^137^Cs maps clearly suggest that the ^137^Cs activity concentration of wood greatly differs among tree species, even in the same areas; according to the different temporal trends, the map of deciduous broadleaf forests shows a drastic increase in wood ^137^Cs activity concentrations, while that for evergreen needleleaf forests shows stable concentrations. Based on the map coupled with the vegetation map, the ^137^Cs activity concentration increased in most areas, and the increase was distinct in the first 10 years but slowed in the next 10 years. This trend mainly corresponds to the temporal trend for the oak trees. Accordingly, the area of more than 50 Bq kg^−1^ spreads over time. However, the southern area in the simulated map shows that the ^137^Cs activity concentration was relatively stable in areas where evergreen needleleaf forests were dominant. The map also demonstrates that even outside of the highly contaminated areas to the northwest of the FDNPP, there will be areas with relatively high ^137^Cs concentrations in the future (the yellow band from northeast to southwest at the centre of the map). However, the map of the trend of the ^137^Cs activity concentration is strongly influenced by the vegetation map, as shown in Fig. 4. In reality, forest stand sizes are much narrower than the resolution of the vegetation map (1 km), and forest stands are often a mixture of evergreen needleleaf and deciduous broadleaf forests.

**Figure 5:**
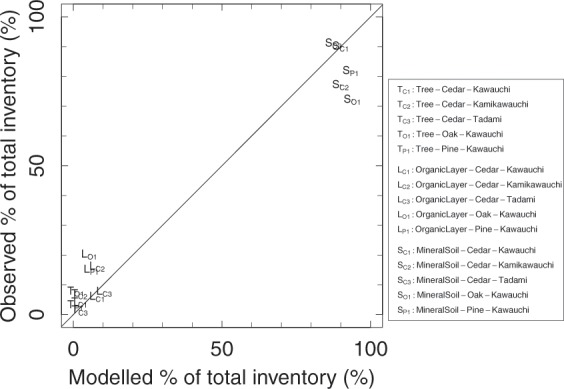
Results of the model validation. The simulated results were validated by comparing the estimated percentages of the total ^137^Cs inventory in 2016/2017 observed data at three cedar sites, one oak site, and one pine forest site. The inventories of ^137^Cs in the tree (T), soil surface organic layer (L), and mineral soil (S) compartments were compared. The subscripts indicate the tree and site identity; C (cedar), O (oak), and P (pine); Kawauchi (1), Kamikawauchi (2), Tadami (3).

**Figure 6:**
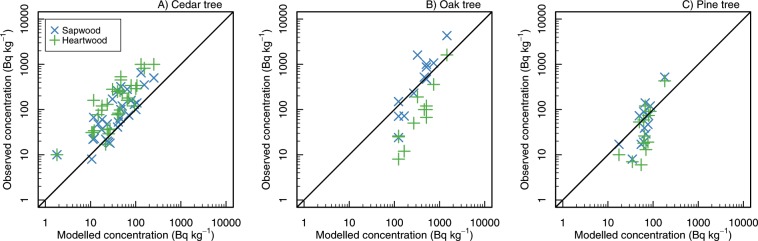
Comparison between the predicted ^137^Cs activity concentrations and those in other studies for cedar (**A**), oak (**B**), and pine (**C**) trees. Please note that the model output for the Otama cedar tree is used for cedar and pine and that for Kawauchi oak is used for oak (see the Methods section). Also please note that the model does not distinguish sapwood and heartwood while the observed data does. Hence, the modelled concentrations are for whole wood. The validation data for cedar and pine are from a report by the Fukushima local government (sampled in 2016), and those for oak are from a journal paper and several governmental reports (sampled in 2012–2015) (see the Methods section).

The simulated values of percentages of the total initial inventory and the wood ^137^Cs activity concentration were validated. The percentages of the total initial inventory simulated were plotted against those observed in other monitoring sites by Forestry and Forest Products Research Institute (FFPRI)^3^ (Fig. 5). The validation indicated that the overall distributions of ^137^Cs within forests were similar among Japanese forests and that the model successfully captured the distributions. The wood ^137^Cs activity concentrations were validated against the monitoring data collected by the Fukushima local government^16^ and data reported in a journal paper^17^ and several governmental reports^18–21^ (Fig. 6, and also see Methods and Supplementary Fig. S4). Please note that the observation data were for sapwood and heartwood, respectively, but the simulated concentrations were for whole wood. This difference is because most of the concentration data for wood measured in Japan came from either sapwood or heartwood, not for whole wood. The modelled ^137^Cs activity concentration was well correlated with those in the observation data. The number of oak trees available for validation was limited; however, the modelled and observed data are of the same magnitude. We further compared the prediction of the ^137^Cs activity and concentration for cedar wood with those from another modelling study (Supplementary Fig. S5; FoRothCs model^11^). The model was parameterized using the data in the same site. The obtained trajectories from our modelling and that of Nishina *et al*. agreed in terms of magnitude, particularly during the observation period, but the difference increased in the latter phase. The estimate by the RIFE1.5 model was stable, while the estimate by the FoRothCs model showed a decreasing trend.

## Discussion

Our modelling analysis aimed to track the overall dynamics of ^137^Cs in forests and to predict the wood contamination levels to guide local people and authorities who reside in the areas and are involved in the forest management of these areas. Using site-scale modelling and regional-scale upscaling based on observation data, our modelling succeeded in providing the informative information as intended. Another purpose of this study was to verify the previous model predictions based on the first 2-year of observation data. The comparison between the latest observation data and previous predictions showed that the simulation of the inventory was successful in predicting the dynamics of ^137^Cs in forest ecosystems, particularly for deciduous broadleaf forests (i.e., the simulations for oak in this study)^10^. On the other hand, although generally well reproduced, the simulation for evergreen needleleaf forest (i.e., the simulation for cedar in this study) failed to predict the long persistence of ^137^Cs in the organic layer (broken purple line in Fig. 1A), and as a result, the model overestimated the migration from the organic layer to the mineral soil. We speculate that the difference in the performance of the model for organic matter compartment between evergreen and deciduous forests was due to the different contamination of needles/leaves caused by the timing of fallout. The fallout occurred prior to leafing, and the leaves of deciduous trees were not contaminated directly by the fallout; in contrast, the needles of the evergreen needleleaf trees were directly contaminated and had high ^137^Cs activity concentration (Fig. 2 in Imamura *et al*. 2017). The contaminated needles could be the source of the internal cycle of ^137^Cs [ref. ^22^]. In deciduous forests, the inventory in the tree canopy was already less than 20% of the total initial inventory, and the major migration from the tree to the soil surface ended. This result means that the key components in the former inventory-based simulations for deciduous broadleaf forests were the organic layer and the mineral soil compartments, and the migration from the tree to the soil surface could slightly affect the organic layer ^137^Cs. On the other hand, the inventory in the tree canopy for evergreen needleleaf trees still retained a certain amount of ^137^Cs in forest ecosystems, and the migration from the tree to the soil floor continued after the second year. The discrepancy of ^137^Cs in the organic layer in the previous prediction may imply that the input from the tree canopy and the output to the mineral soils were not well represented in the former model. In addition, the longer persistence of ^137^Cs may be partly due to the uptake by or retention by fungi^23^. The soil surface organic layer is the key interface between the tree and the mineral soil, and the ^137^Cs in the organic layer is more mobile than that in the mineral soil; therefore, monitoring together with background process research in the organic layer is necessary.

However, when we look at the overall trends, the trends were the same between the previous (based on 2 years of data) and new (based on 6–7 years of data) predictions. In the preliminary application before parameterization using data observed in Japan, the model with default (i.e., geometric mean parameter values from Chernobyl studies) failed to reproduce the first two-year ^137^Cs dynamics in forest ecosystems in Japan^10^. This result implies that the model cannot be parameterized using data obtained in very different forests, as the model will not necessarily work well in other forests; however, once the model is parameterized using site specific data, even using a limited period of time-dependent data at the site, the model can adequately capture the overall changes in ^137^Cs in forest ecosystems^24^. The importance of updating the default parameters using observation data was also reported in other modelling studies in Fukushima^11^. Most likely, as occurred after the FDNPP accident, in the case of a nuclear accident elsewhere in the future, there will likely be only limited data available for modelling despite the strong need to apply a model to help understand the dynamics and obtain predictions necessary to build countermeasures. Hence, the above result is an important lesson from the iterative parameterization and verification/validation demonstrated in the present and previous studies.

Our model analysis revealed that the annual flux of ^137^Cs from the tree to the soil surface very quickly decreased from more than 10% to below 1% of the total initial inventory in the first 5–10 years, suggesting that the ^137^Cs migration within forests rapidly converged and forest ecosystems worked as ^137^Cs stabilizers and reservoirs. At the same time, the analysis suggests that, after the initial rapid drop of the circulation rate, although below 1% of the total initial inventory, ^137^Cs continues to circulate within forests and affect the ^137^Cs activity concentration of tree organs. Our modelling, however, does not assume the possible fixation of ^137^Cs by clay minerals in soil; hence, the circulation in the future may become slower than that expected in this modelling approach.

The observed time dependency of ^137^Cs activity concentration in wood differed among tree species. The concentration of oak trees showed a rapid increase in the first 3 years after the fallout and that for cedar did not show such a substantial increase in the observation period. Perhaps this difference is caused by the different physiological nature among tree species as well as by different initial contamination patterns. Although the model does not have detailed processes within the tree body, the parameterized model successfully reproduces the distinct difference in time-dependency in ^137^Cs activity concentrations among tree species.

The aggregated transfer factor values (*T*_ag_: the ratio of ^137^Cs activity concentration in plant organs to the total soil inventory (soil surface organic layer + mineral soil)) for wood were evaluated at the end of the simulation period (e.g., 20 years after the accident). The *T*_ag_ values were 2.2 × 10^−4^ m^2^ kg^−1^ DW (dry weight) (1.3 × 10^−4^ m^2^ kg^−1^ DW in the Kawauchi site) for cedar, 9.6 × 10^−4^ m^2^ kg^−1^ DW (5.2 × 10^–3^ m^2^ kg^−1^ DW in Kawauchi) for oak, and 2.9 × 10^−5^ m^2^ kg^−1^ DW for pine. Although the *T*_ag_ values obtained in the early phase after the accident were generally strongly influenced by direct deposition, the factor in the latter phase, which was derived from the 20-year simulation in this study, should be less influenced. The *T*_ag_ values for wood observed in Europe were compiled and reported in Calmon *et al*.^25^ and in International Atomic Energy Agency documents^26,27^. The geometric mean of wood *T*_ag_ values observed in European countries after the Chernobyl accident^25^ were 1.5 × 10^−3^ m^2^ kg^−1^ DW (N = 31, min = 1.1 × 10^−4^, max = 2.1 × 10^−2^) for coniferous trees and 3.5 × 10^−4^ m^2^ kg^−1^ DW (N = 12, min = 1.0 × 10^−5^, max = 3.8 × 10^−3^) for deciduous trees, and the overall geometric mean was 1.0 × 10^−3^ m^2^ kg^−1^ (N = 43, min = 1.0 × 10^−5^, max = 2.1 × 10^−2^). The *T*_ag_ values derived from our model analysis were the same magnitude as those derived from the Chernobyl compilation. The values for cedar seemed to be smaller than the value for coniferous trees reported in European countries and the values for oak seemed to be larger than the values for deciduous trees reported in European countries. However, the available data were limited; thus, they were not conclusive.

The structure of the developed model is relatively simpler than other detailed models. Nevertheless, this study clearly proves that the model works well and is a reliable tool that can be used to predict the future of ^137^Cs in forest ecosystems. However, some potential limitations, which are sources of uncertainty, should be stated. Observations revealed that the ^137^Cs activity concentration of sapwood and heartwood differed, particularly with that of cedar^15^. The prediction of ^137^Cs activity concentration in wood may require explicitly dividing the sapwood and heartwood when the concentration in sapwood and heartwood differ considerably. Another limitation is the simple vertical process used in this modelling framework. The soil surface was divided into litter (fresh dead organic material) and organic soil (humified organic layer), but the mineral soil consisted of one component. This one-component model was reasonable because most ^137^Cs in the soil was distributed in the shallow layer (<0.05 m) and because model simplification was required. However, the vertical distributions of ^137^Cs within the soil and spatio-temporal changes in the mobility of ^137^Cs in the soil are important factors in terms of quantifying the uptake of ^137^Cs by trees via roots. For the uptake process, specific soil properties (e.g., minerals^28^ and potassium^29,30^) are also influential. For instance, the different concentrations of oak between Otama and Kawauchi may be attributable to the soil properties. In addition, as mentioned above, the lack of processes of the possible fixation of ^137^Cs by clay minerals in soil in this model may result in overestimation of root uptake. For the ^137^Cs dynamics within the tree body, the seasonality of the ^137^Cs activity concentration in the tree organs (e.g., needles/leaves) was not simulated in the model. Observations with intense time intervals (e.g., monthly) suggest that the ^137^Cs activity concentration shows a clear seasonal trend. Unfortunately, data from the monitoring efforts used in this study were not designed to capture seasonality; rather, the monitoring approach was designed to capture the overall annual dynamics within forests. However, when detailed dynamics within the tree body are simulated, the detailed translocation and dynamics within the tree body must be incorporated into the model. Additionally, although discharge of ^137^Cs from forest catchments via stream water, which was mostly driven by suspended sediments, was detected in observations, the total annual discharge reported was less than 0.3% of the total deposition of the catchment^31,32^. The model did not incorporate the process of discharge from forest ecosystems but focused only on the internal cycle of ^137^Cs in forests.

The uncertainty of the spatio-temporal prediction of the ^137^Cs activity concentration in wood should also be discussed. In addition to the difference among tree species, as shown in the difference between the Otama and Kawauchi sites and in the comparison with the data observed at other sites, the variation in the ^137^Cs activity concentration in wood for a tree species has large variation, even within a similar deposition level. Another issue is our conservative estimation. The trajectories of higher concentrations were adopted for the spatial evaluation in this study, and we do not deny the possibility that the future concentration may be lower than the concentration we predicted. A possible explanation is that the possible fixation of ^137^Cs by minerals may lower the ^137^Cs uptake by trees (but also see Manaka *et al*. 2019 and Koarashi *et al*. 2019)^28,33^. The model modification performed in this study may affect the trajectory; in this study, the source of root uptake was changed from the organic compartment to the mineral compartment because the organic layer is very thin in Japan, and we believe this approach is more reasonable and provides more conservative estimates in terms of continued root uptake. The ^137^Cs inventory in the organic compartment will decrease with time, while that in the mineral compartment will persist and become the largest; hence, the assumption of the source of ^137^Cs affects the future root uptake of ^137^Cs. The stand age may also affect the trajectory. Our modelling was parameterized using the forest stand where trees were planted before the accident, so the trajectory may not be applicable to trees that were planted after the accident.

Even with the potential limitations due to simplification and uncertainty, our model analysis showed good performance against the observation data and provided insightful information that can be used by the public and authorities involved in the management of contaminated forests. As the previous prediction published in 2013 was verified with the latest data obtained in this study, the new model prediction must be iteratively updated and verified using the latest data; this approach will help evaluate and enhance the ability of radionuclide dynamics models. Finally, we emphasize that the continued monitoring of ^137^Cs dynamics in forests is necessary.

## Methods

### The model

In the present work, we applied a modified version of the RIFE1 model (RIFE1.5; Supplementary Fig. S6A,B). The original RIFE1 (Radionuclides In Forest Ecosystems) model was developed by Shaw and Belli^24,34,35^ and was used in the previous prediction^9^. The RIFE1 model consists of 5 compartments (external tree, internal tree, litter, organic soil, and mineral soil), and these compartments are linked by radionuclide fluxes, which are described as a first-order rate process. The RIFE1 model was developed to provide forecasts of radionuclide behaviour after fallout to decision makers and designed to run with limited data; hence, the model structure is relatively simple. As in the previous prediction, we assumed the leaf, branch, and bark as the external tree compartment, and the wood (sapwood and heartwood) as the internal tree compartment.

In the modified RIFE1 model, the uptake flux from the soil to the external tree compartment (e.g., leaf, branch, and bark compartments combined) was included. In the original RIFE1 model, no uptake from root to the external tree compartment was assumed, but in this model, we classified the needle/leaf, branch, and bark components as external, and we assumed including root uptake in this compartment was necessary (*Q*_5_ to *Q*_1_ and *Q*_6_ flux in Supplementary Fig. S6A,B). We further assumed that the mineral soil compartment was the source of root uptake (the original model assumes the organic layer is the source).

We also developed a dual tree-species version of the RIFE1 model (Supplementary Fig. S6B). In some of the monitoring sites, the forests consist of two dominant species, e.g., oak and pine, and the trends of the ^137^Cs activity concentration in wood differ among the tree species. In the preliminary simulations, we found that the single tree species model could be used to simulate the inventory within forests because the inventory in tree compartments is very small; however, to predict the ^137^Cs activity concentration in wood, we needed to distinguish the two tree species. We applied the single tree-species version of the model to the forest with a single dominant tree species, and we applied the model with two tree species to mixed forests.

The model equations for the single tree species version of the new model are as follows:

$${\rm{d}}{Q}_{1}/{\rm{d}}t={I}_{1}-({k}_{1}+{k}_{4}+\lambda ){Q}_{1}+{k}_{8}{Q}_{5}$$

$${\rm{d}}{Q}_{2}/{\rm{d}}t=-\,({k}_{2}+\lambda ){Q}_{2}+{k}_{3}{Q}_{5}$$

$${\rm{d}}{Q}_{3}/{\rm{d}}t={I}_{3}-({k}_{5}+\lambda ){Q}_{3}+{k}_{4}{Q}_{1}+{k}_{2}{Q}_{2}$$

$${\rm{d}}{Q}_{4}/{\rm{d}}t={I}_{4}-({k}_{6}+\lambda ){Q}_{4}+{k}_{5}{Q}_{3}$$

$${\rm{d}}{Q}_{5}/{\rm{d}}t={I}_{5}-({k}_{8}+{k}_{7}+{k}_{3}+\lambda ){Q}_{5}+{k}_{6}{Q}_{4}$$

where *Q*_1_ is the inventory of ^137^Cs within needles/leaves, branches, and bark, and *Q*_2_, *Q*_3_, *Q*_4_, and *Q*_5_ are those within whole wood, litter (fresh dead leaves on the soil surface), soil surface organic soil (decomposed organic matter on the soil surface; F (partially decomposed) and H (well-humified) layers combined), and mineral soil compartment, respectively (Bq m^–2^). *k*_i_ and *λ*_i_ are the transfer rate coefficients between compartments (yr^–1^) and the rate constant of radioactive decay (yr^–1^) for the ^137^Cs, respectively. *t* is the time (yr), and *I*_i_ is the input of ^137^Cs to each component at the time of fallout (but please see the Data section below). For the dual tree species model, the equations are as follows:

$${\rm{d}}{Q}_{1}/{\rm{d}}t={I}_{1}-({k}_{1}+{k}_{4}+\lambda ){Q}_{1}+{k}_{8}\,{Q}_{5}$$

$${\rm{d}}{Q}_{2}/{\rm{d}}t=-\,({k}_{2}+\lambda ){Q}_{2}+{k}_{3}{Q}_{5}$$

$${\rm{d}}{Q}_{3}/{\rm{d}}t={I}_{3}-({k}_{5}+\lambda ){Q}_{3}+{k}_{4}{Q}_{1}+{k}_{2}{Q}_{2}+{k}_{12}{Q}_{6}+{k}_{10}{Q}_{7}$$

$${\rm{d}}{Q}_{4}/{\rm{d}}t={I}_{4}-({k}_{6}+\lambda ){Q}_{4}+{k}_{5}{Q}_{3}$$

$${\rm{d}}{Q}_{5}/{\rm{d}}t={I}_{5}-({k}_{13}+{k}_{11}+{k}_{8}+{k}_{7}+{k}_{3}+\lambda ){Q}_{5}+{k}_{6}{Q}_{4}$$

$${\rm{d}}{Q}_{6}/{\rm{d}}t={I}_{6}-({k}_{9}+{k}_{12}+\lambda ){Q}_{6}+{k}_{13}{Q}_{5}$$

$${\rm{d}}{Q}_{7}/{\rm{d}}t=-\,({k}_{10}+\lambda ){Q}_{7}+{k}_{11}{Q}_{5}$$

where *Q*_6_ and *Q*_7_ are the inventories of ^137^Cs for the sum of needles/leaves, branches, and bark inventories and the inventory for wood of the second tree species, respectively. The model is applicable to other nuclides if the rate constant of radioactive decay is changed to reflect the radionuclide being considered and if other parameters are determined based on the radionuclide being considered using the observation data.

The transfer rate coefficient and the rate constant of the radioactive decay were described using the half-lives (*T*_h_) based on the following relationship:

$$k(\lambda )=\,\mathrm{ln}(2)/{T}_{{\rm{h}}.}$$

The RIFE1.5 model simulates the ^137^Cs inventories between structural components within forest ecosystems. In the new modelling framework, we simulated the ^137^Cs activity concentration in wood by dividing the ^137^Cs inventory in wood (Bq m^−2^) by the wood volume (kg m^−3^). The growth curve of wood biomass was estimated using data from the National Forest Inventory^36^. The wood biomass data, including the stand ages of cedar, oak, and pine observed in Fukushima prefecture and neighbouring prefectures (Tochigi, Ibaraki, Miyagi prefectures), were extracted from the database and were fitted with an exponential growth curve (the monomolecular growth model or the Mitscherlich model):

$$B=a\cdot {b}^{\exp (-c\cdot Age)},$$

where *a*, *b*, and *c* are constants, and *B* and *Age* are the wood biomass (kg m^−2^) and the stand age (yr), respectively. When fitting this function to the data, to correct the bias in the data distribution between the different stand age classes for the conversion of the best parameter estimation, we resampled data from 25-year-old age intervals and fitted the function to the resampled data. The function curve was then scaled with the observed wood biomass at each site (see below), and the biomass growth after the observation period was estimated.

The source code for the basic simulation is available in the Supplementary Information.

### Parameterization

The model was parameterized using the data observed in the 6–7 year period after the FDNPP accident (see Data section). A Bayesian calibration scheme, which is an optimization scheme that uses Monte Carlo sampling, was used to estimate the best transfer parameters (*k*_i_) (Supplementary Table S1). Details about the Bayesian calibration scheme are described elsewhere^37^. We conducted 500,000 iterations of sampling and discarded the first 100,000 iterations as burn-in to avoid the influence of the initial values. A standard deviation of 30% of the mean value was assumed for each compartment for the error function in this study. The maximum a posteriori (MAP) estimates were used as the best-fit parameters.

### Data

For the simulation of temporal changes in the inventory data, we parameterized the model using the inventory data on the ^137^Cs observed in the study site^3,5,15,38^ in Otama village (37°34′N, 140°18′E) of Fukushima prefecture. There are three monitoring sites in the Otama site: 1) sugi cedar forest (*Cryptomeria japonica*: corresponding to evergreen needleleaf forest in the previous prediction), 2) konara oak (*Quercus serrata*) dominant mixed forest with red pine (*Pinus densiflora*) (corresponding to deciduous broadleaf forests in the previous study), and 3) red pine dominant mixed forest with konara oak. The simulation for red pine dominant mixed forest with oak (site 3) was newly incorporated in this study. The observed ^137^Cs distributions were converted to the percentage of the total initial inventory and used in this study. The data observed in August-September 2011 were used as the initial condition for this simulation (hence, the *I*i values were assumed to be zero in this study, and the initial trap of the fallout and the migration of ^137^Cs in the first 5 months was not in this simulation). The depth of the litter and organic soil for *k*_5_ and *k*_6_ were, as in the previous study, set to 0.017 and 0.012 m, respectively, and these values were measured in Fukushima in the national soil carbon inventory project. In the field measurement data, the portions of ^137^Cs in the litter and organic soil were not distinguished; we assumed that 20% of the observed ^137^Cs was retained in the litter (L layer) and 80% was retained was in the organic soil (F and H layers) to prepare the initial conditions for *Q*_3_ and *Q*_4_. We set the mineral soil layer thickness at 0.2 m for *k*_7_, as in our field observation monitoring, but we assumed no downward flux from *Q*_5_ (*k*_7_ = 0). In this study, we simulated the dynamics of ^137^Cs in 5 compartments for the single tree species model and 7 compartments for the dual tree species model (see the model section). For clarity, the total ^137^Cs in the tree components (*Q*_1_ and *Q*_2_, and *Q*_6_ and *Q*_7_; the inventories of ^137^Cs for the sum of needles/leaves, branches, and bark inventories and the inventory for wood, respectively, in RIFE1.5), the total in the soil surface organic layer (*Q*_3_ and *Q*_4_; litter and organic soil compartments), and the total in the mineral soil (*Q*_5_; mineral soil in the model) are shown. We ran the model at monthly time steps (i.e., 12 time steps/year) and obtained the yearly output. The observed ^137^Cs activity concentrations for wood were converted to normalized concentrations based on the total initial inventory. Because the observed total inventory fluctuated, the average in the observation period was used to normalize the concentrations.

For the calibration and the simulation of temporal changes in the ^137^Cs activity concentration for cedar and oak wood, we used the observation data obtained in the Otama and Kawauchi sites^3,5,15,38^ (Kawauchi site: 37°17′N, 140°48′E) in Fukushima prefecture. The activity concentrations were also normalized using the total initial inventory.

The single tree species model was used for the cedar forest in Otama, and the dual tree species model was used for the other forests in both Otama and Kawauchi sites.

### Spatial application

To provide a conservative estimate, we used the model output for cedar trees at the Otama site as the value for the evergreen needleleaf forest and that for oak trees at the Kawauchi site as the value for the deciduous broadleaf forest; these sites were chosen because they have a higher future activity concentration in the model output. In the preliminary application and validation using the other data, we found that the simulation for oak based on the Otama site underestimated the ^137^Cs activity concentration in wood (Supplementary Fig. S4B). Instead, we confirmed that the activity concentration based on the Kawauchi site corresponded well to the other observation data (Fig. 6B). Therefore, for the simulation of the oak wood ^137^Cs activity concentration, we used the model parameterized with data obtained at the Kawauchi site. Furthermore, the pine wood ^137^Cs activity concentration based on pine trees at the Otama site underestimated the other observation data (Supplementary Fig. S4C). We do not have other long-term monitoring sites for pine; therefore, we could not determine the reason for the difference and could not re-parameterize the model to match the simulated concentrations with those in the Fukushima local governmental data. Therefore, we used the simulation results for cedar to predict the ^137^Cs activity concentration for evergreen needleleaf forests, and the simulation results for cedar were well correlated with the observation data for pine (Fig. 6C).

Then, we combined the simulated dynamics with an air-borne survey map of the ^137^Cs distribution^39^ and a vegetation map^40^. The resolution of the ^137^Cs map was approximately 250 m. The evergreen needleleaf forest and deciduous broadleaf forest were distinguished in the vegetation map. The resolution of the vegetation map was approximately 1 km, and each 1 km grid cell had a vegetation type. We multiplied the ^137^Cs deposition in a grid, as estimated by the air-borne survey (Bq m^−2^), with our simulated ^137^Cs activity concentrations in wood for the tree species, and we estimated the time dependence of the ^137^Cs activity concentration in wood for each grid. In other words, in a grid of an evergreen needleleaf forest, the concentration for wood was calculated as the air-borne survey based inventory × the normalized concentration for wood for cedar.

## Supplementary Information

Supplementary Information.

Supplementary Information2.
