# Supplementary material for: New predictions of 137Cs dynamics in forests after the Fukushima nuclear accident
Source: Sci Rep. 2020 Jan 8;10:29. doi: 10.1038/s41598-019-56800-5 (PMC6949301; doi:10.1038/s41598-019-56800-5)
Supplement: Supplementary file 2 — Supplementary Information2. [file 41598_2019_56800_MOESM2_ESM.pdf]

# New predictions of $^{137}Cs$ dynamics in forests after the Fukushima nuclear accident

**Shoji Hashimoto, Naohiro Imamura, Shinji Kaneko, Masabumi Komatsu, Toshiya Matsuura, Kazuya Nishina, Shinta Ohashi**

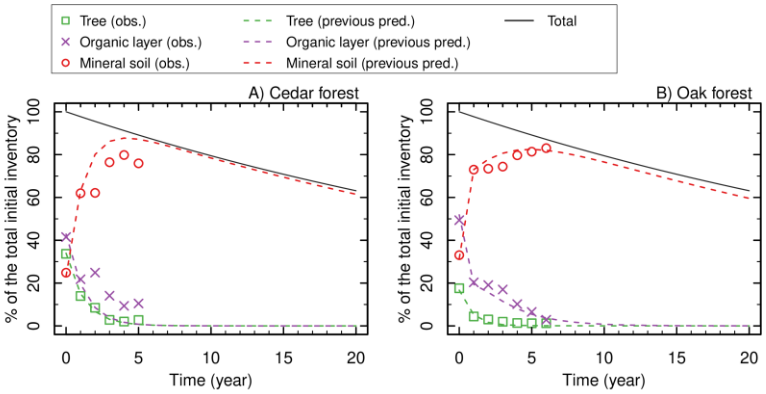


Fig. S1: Temporal changes in the $^{137}$Cs distributions in cedar (A) and oak (B) forests of the Otama site. The dashed lines were from the study of Hashimoto et al. 2013 and are based on the observation data collected in 2011 and 2012. Please note that both $^{134}$Cs and $^{137}$Cs were simulated in the previous study, but the results for $^{137}$Cs were verified here.

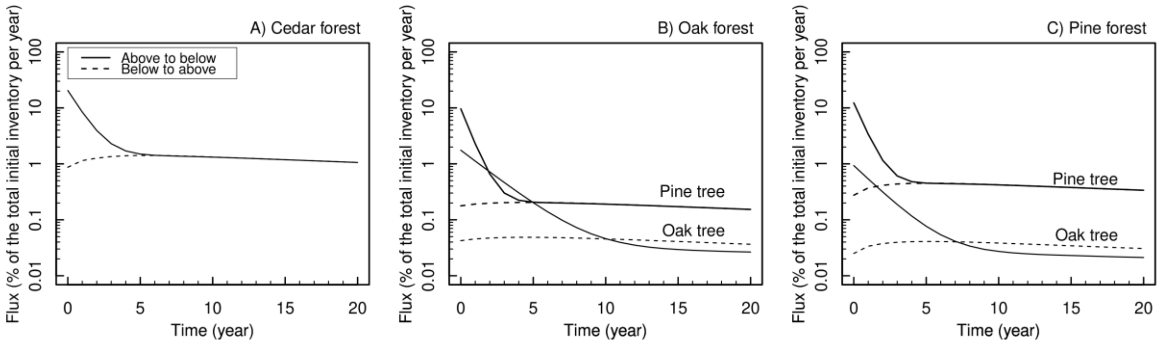


Fig. S2: Temporal changes in the $^{137}Cs$ flux from tree to soil (above to below flux; solid lines) and tree uptake (below to above flux; dashed lines) flux in cedar forests (A; similar to Fig. 2) and in mixed forests of oak and pine trees (B, C). In this figure, the fluxes for pine and oak trees were distinguished.

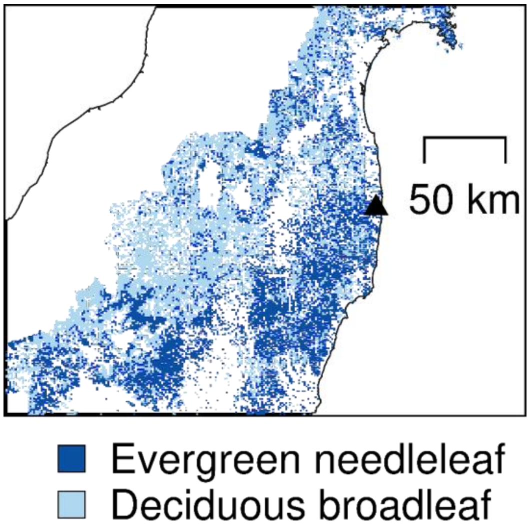


Fig. S3: The vegetation map used in this study, in a resolution of 1 km. The map was created using the Generic Mapping Tools version 5 (http://gmt.soest.hawaii.edu/).

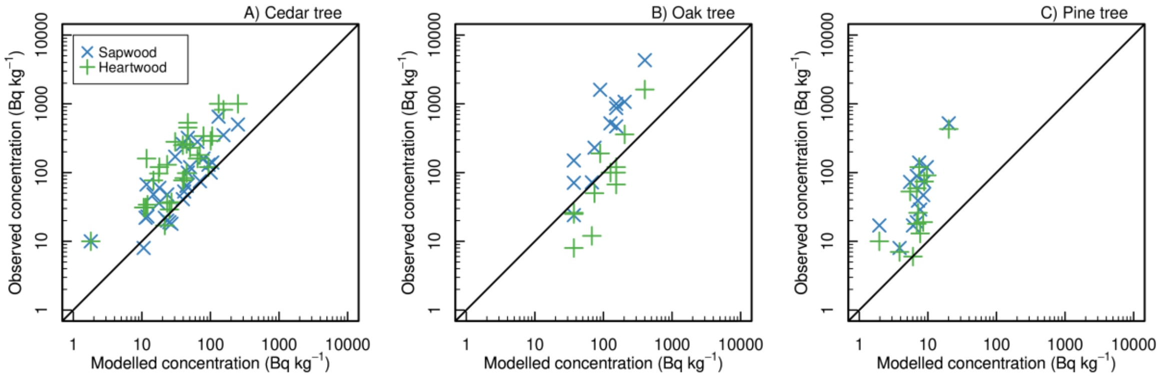


Figure S4: Comparison between the predicted $^{137}$Cs activity concentration, based on the parameterization with the data of the Otama site, and concentrations reported in other studies for cedar (A), oak (B), and pine (C) trees. The modeled concentrations are for the whole wood, because the developed model does not distinguish the sapwood and heartwood. The validation data for cedar and pine were obtained from a report of the Fukushima local government (2016), and those for oak are from a journal paper and several governmental reports (sampled in 2012–2015) (see the Methods section).

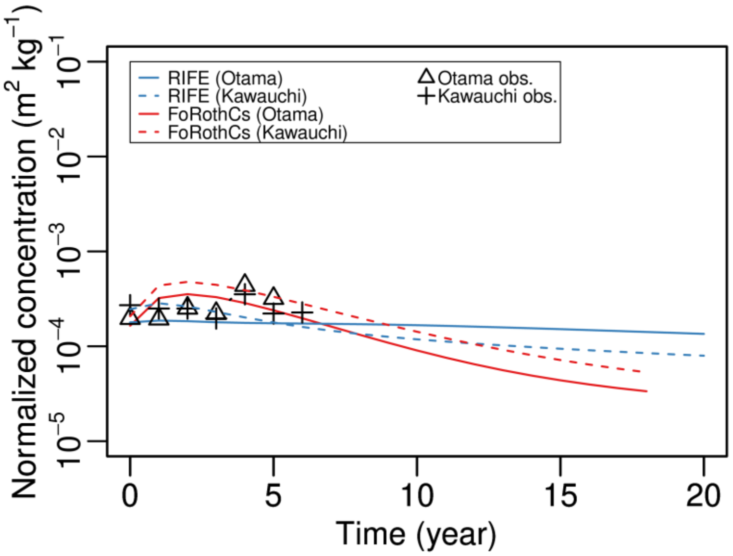


Fig. S5: Comparison between the $^{137}Cs$ activity concentrations estimated in the current study using the RIFE model (blue lines) and the concentrations obtained during the study of Nishina et al., using the FoRothCs model (red lines)[11]. The trajectories of both studies were of the same magnitude particularly during the observation period, but significant variations were noticed in the latter phase. The estimation by the RIFE1.5 model was stable, whereas the estimation by the FoRothCs model exhibited a decreasing trend.

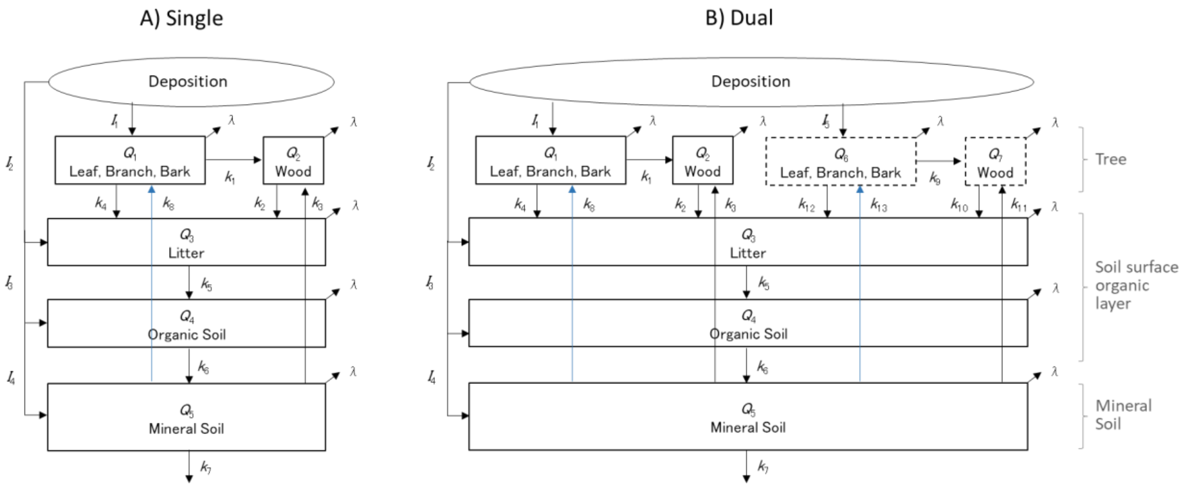


Fig. S6: Diagram of the model structures. Single-tree species model (A) and dual-tree species model (B). Newly incorporated fluxes are presented in blue.

Table S1. Parameters of the RIFE1.5 model. Please note that the parameters are presented as half-lives in this table and that the values for $k_5$, $k_6$, and $k_7$ are multiplied by the thickness of the relevant soil layer, when used. The dual-tree species model was applied to oak and pine forests in Otama and to cedar and oak forests in Kawauchi. $Q_1$ and $Q_2$ corresponded to pine or cedar and $Q_6$ and $Q_7$ to oak (Fig. S4).

| Site | Otama | | | Kawauchi |
|---|---|---|---|---|
| Forest | Cedar | Oak (+Pine) | Pine (+Oak) | Cedar (+Oak) |
| Parameter | Half-life time (yr or yr $m^{-1}$) | | | |
| $k_1$ | 300.6 | 427.5 | 449.3 | 237.0 |
| $k_2$ | 2.355 | 0.981 | 0.818 | 2.719 |
| $k_3$ | 3246.5 | 19935.7 | 18312.0 | 11439.0 |
| $k_4$ | 0.766 | 0.498 | 0.545 | 0.744 |
| $k_5$ | 87.8 | 120.4 | 97.3 | 63.0 |
| $k_6$ | 18.60 | 3.27 | 50.28 | 13.54 |
| $k_7$ | - | - | - | - |
| $k_8$ | 203.9 | 1493.4 | 647.7 | 3017.8 |
| $k_9$ | - | 299.2 | 321.9 | 383.7 |
| $k_{10}$ | - | 22.72 | 40.69 | 16.66 |
| $k_{11}$ | - | 10889.8 | 15876.1 | 2414.0 |
| $k_{12}$ | - | 1.64 | 1.312 | 3.009 |
| $k_{13}$ | - | 12589.2 | 12168.9 | 10994.1 |
| $\lambda$ ($^{137}Cs$) | 30.17 | | | |

R code for the RIFE1.5 model (the basic single-tree species model): see the next Supplementary code.
